# Primary Pulmonary Lymphoma Presenting with Superior Vena Cava Syndrome in a Young Female

**DOI:** 10.1155/2017/1937107

**Published:** 2017-08-08

**Authors:** Divya Salhan, Prakash Verma, Tun Win Naing, Ebad Ur Rehman, Saroj Kandel, Danillo Enriquez, Joseph Quist, Frances Schmidt

**Affiliations:** Interfaith Medical Centre, Brooklyn, NY, USA

## Abstract

Primary Pulmonary Diffuse Large B Cell Lymphoma (PPDLBCL) is an extremely rare entity, which exhibits an aggressive behavior by compressing local blood vessels. It represents only 0.04% of all lymphoma cases and is extremely rare in young age. We present a case of a primary pulmonary lymphoma with superior vena cava syndrome (SVCS) in a young female. 27-year-old African American female presented with fever, cough, and facial puffiness for 2 weeks and unintentional weight loss. Chest examination showed decreased breath sounds and dullness on percussion on right side. Labs were normal except for mild leukocytosis, high lactate, and lactate dehydrogenase. Chest X-ray showed a large right side infiltrate with pleural effusion but chest CT showed 10 × 14 × 16 cm mass in the right lung without hilar and mediastinal lymphadenopathy. CT guided biopsy of the right lung mass was done and large B cell lymphoma was diagnosed. She received “involved field radiation” because of the bulky tumor size and superior vena cava involvement prior to R-CHOP to which she responded well. PPDLBCL should be considered as one of the differentials in a young patient with a large lung mass, which needs timely diagnosis and management.

## 1. Case Presentation

27-year-old African American female with no significant past medical history presented with fever, productive cough, and significant facial puffiness for the past 15 days. She also reported night sweats and unintentional weight loss of 15 lbs. in the past 1 month. She visited emergency twice in the past one month and was also prescribed a respiratory quinolone for 7 days by her primary medical doctor but her symptoms did not improve significantly. Patient denies any similar episode prior to this presentation. No significant family history was found. She is a nonsmoker and emigrated from Ghana in childhood.

Physical examination was remarkable for mild respiratory distress, visible facial puffiness, decreased breath sounds, dullness on percussion, and increased tactile fremitus on right side of the chest.

Vital signs were within acceptable limits except for tachycardia (127) and tachypnea (22) on the day of presentation. Laboratory investigations revealed a normal complete blood count with hemoglobin of 12 g/dL, white blood cells of 8300/uL, and platelets of 424000/uL. Other significant laboratory data included elevated lactate level of 3.1 and lactate dehydrogenase of 1475 IU/L.

**Figure 1:**
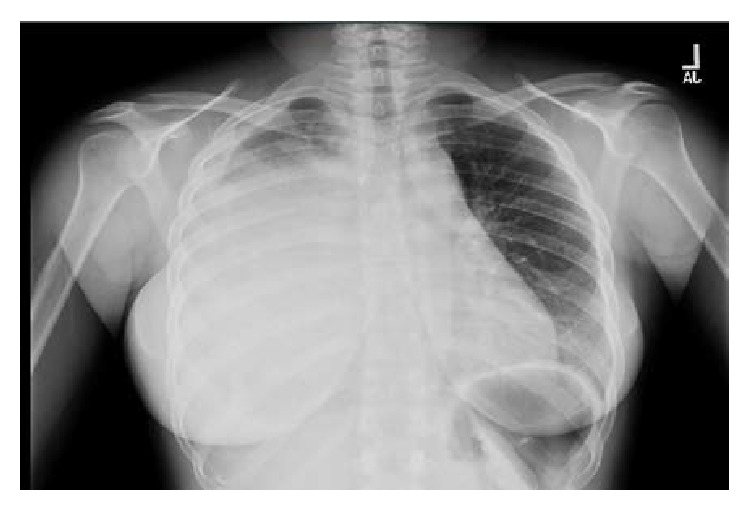
CXR on admission showing large right-sided infiltrate.

**Figure 2:**
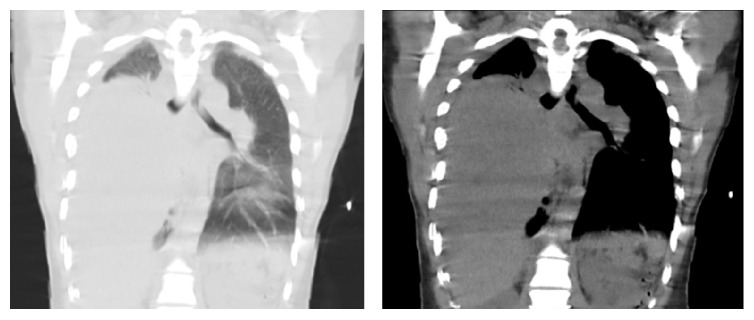
Initial plain chest CT showing the mass.

**Figure 3:**
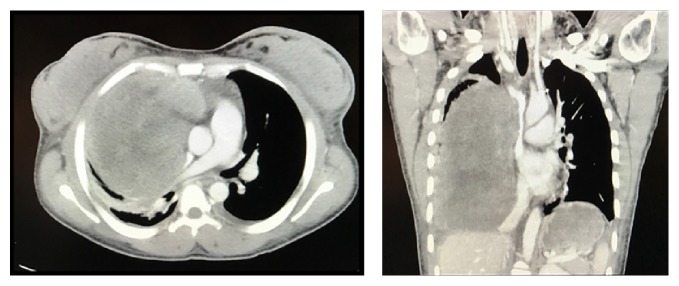
Chest CT showing the superior vena cava obstruction.

**Figure 4:**
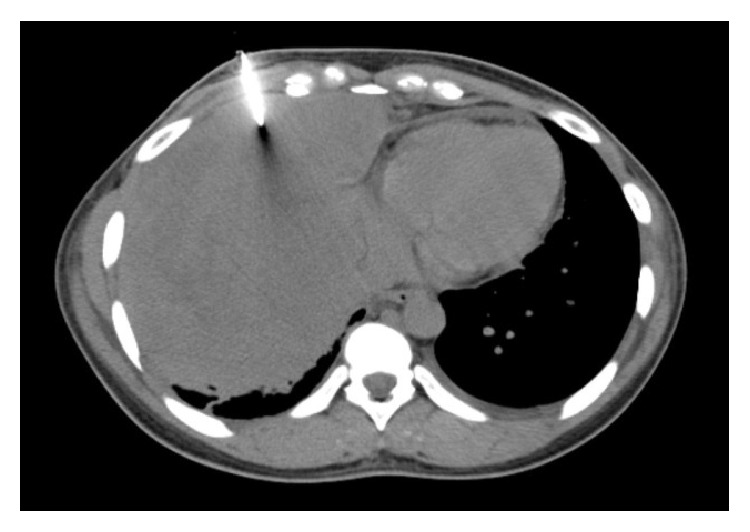
CT guided biopsy of mass.

Chest X-ray revealed a large right-sided infiltrate with pleural effusion (Figure 1). She was admitted to the Intensive Care Unit with airborne precautions with working diagnosis of bacterial pneumonia to rule out pulmonary tuberculosis as she reported fever and night sweats for one month. Three sets of sputum samples were collected on three different occasions for acid-fast bacilli testing which were reported negative. Later chest CT without contrast revealed 10 × 14 × 16 cm mass in the right lung without hilar lymphadenopathy, small pericardial effusion, bilateral pleural effusions, and compressive atelectasis of the right lung (Figure 2) and possible differentials were pleural fibroma, sarcoma, pulmonary pleural blastoma, and primary pulmonary lymphoma (no palpable lymph nodes). The patient subsequently underwent chest CT with contrast for possible superior vena cava obstruction (Figure 3) and CT guided core biopsy of the right lung mass for tissue diagnosis (Figure 4).

**Figure 5:**
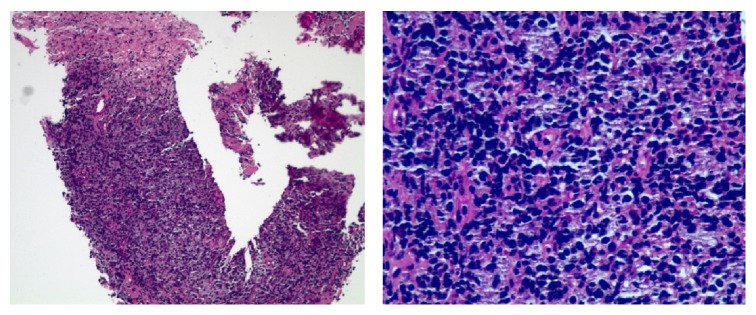
Histopathology slide showing large amount of lymphocytes.

The pathology report revealed atypical B cell rich lymphoid infiltrate consistent with large B cell lymphoma (Figure 5). Immunohistochemistry showed expression of vimentin, leukocyte common antigen (LCA), cluster of differentiation 20 (CD20), CD79a, and CD99 but not of CD3, CD5, CD10, or thyroid transcription factor- (TTF-) 1. Based on these findings, mature large B cell lymphoma was diagnosed.

**Figure 6:**
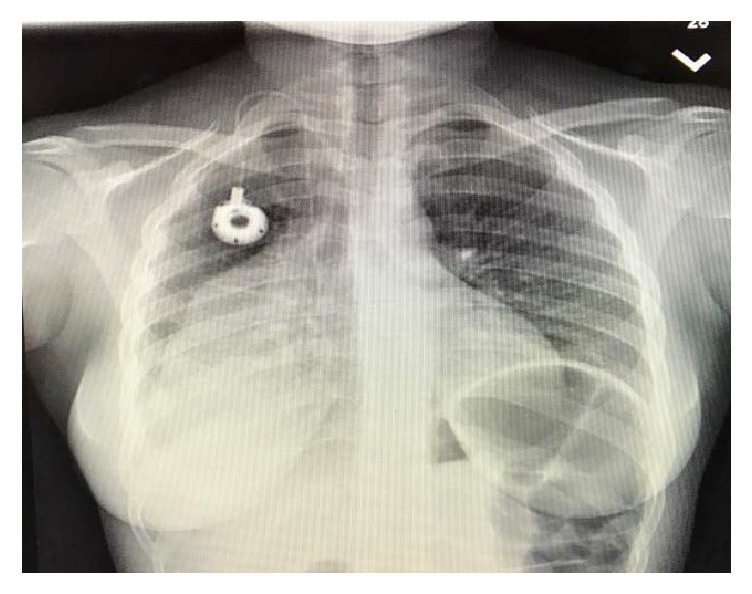
CXR after chemotherapy showing decrease in size of right lung mass.

Initially, patient received “involved field radiation” for two days because of the bulky tumor size and superior vena cava involvement. Patient was started on R-CHOP (rituximab + cyclophosphamide, doxorubicin, vincristine, and prednisone). She responded well to the treatment and tumor was shrunken down on repeated CXR (Figure 6).

## 2. Discussion

Primary pulmonary lymphoma (PPL) is defined as clonal lymphoid proliferation in which single or both lungs are involved without extrapulmonary manifestation at the time of diagnosis or within 3 months of diagnosis. Diffuse large B cell lymphoma (DLBCL) represents 30% of all lymphomas, but extranodal pulmonary DLBCL only accounts for 0.04% of all lymphomas.

Incidence of DLBCL depends on age and ethnicity. It is more common among elderly males, median age of 64 years, and Caucasians Americans but occurs at younger age in African Americans. It is also associated with weak immune system like HIV positive population, Epstein Barr viruses, and positive family history [1]. Our patient is peculiar as she was found to have pulmonary DLBCL at a very young age with no predisposing risk factors mentioned above.

Superior vena cava syndrome (SVCS) is reported to develop in only 2 to 4% of cases of pulmonary non-Hodgkin lymphoma (NHL) [2]. Among pulmonary NHLs, DLBCLs are the most common subtypes that are associated with SVC syndrome. As per literature, very few cases, only about 7% of DLBCLs have SVC syndrome at initial presentation [3]. Our patient presented with SVC involvement, which led us to have an immediate tissue diagnosis.

Our case presented with elevated lactic acid level, which can be multifactorial in lymphomas. As per literature, aggressive tumor cells tend to divide quickly which outgrow their blood supply creating relative hypoxia within the tumor bed leading to anaerobic glycolysis with activation of enzyme lactate dehydrogenase and production of lactate. Other mechanisms that explain lactic academia in malignancy include overexpression of hexokinase and abnormal IGF signaling in malignant cells leading to upregulation of glycolysis and the unique property of cancer cells relying on glycolysis even in the presence of oxygen (Warburg effect) [4–6].

As per study of Lewis et al. for CT findings for lymphoma of the lung, the most common finding was mass-like consolidations ranging from 1 to 8 cm followed by nodules less than 1 cm [7]. On review of literature, 50%–90% of primary pulmonary lymphoma was a localized alveolar opacity with a diameter of <5 cm and blurred or well-defined contours. In our case, patient's lung mass is the largest pulmonary DLBCL ever reported. It was about 10 × 14 × 16 cm mass almost occupying the entire right lung.

Tissue diagnosis with immunohistochemistry is not only the mainstay in diagnosis of lymphomas but also useful for predicting the types of treatment that might be successful and for monitoring the effectiveness of treatment. Our patient's immunohistochemistry was positive for CD20, CD79a, and CD99. It has been studied that CD 99 positive DLBCL has a better 2-year survival value than negative ones [8]. Moreover, the rituximab treatment is more effective in all B cell lymphomas including DLBCL because of their stronger CD20 expression [9, 10]. Vimentin although not characteristic of lymphomas has been reported to be positive in other case reports of PPDLBCL [11]. Observation study done by Tamaru et al. showed some association of vimentin with B cell lymphomas. It is not clearly understood but twelve of the 61 B cell lymphomas were positive for vimentin [12].

Further management of non-Hodgkin lymphomas depends on the International Prognostic Index (IPI) which includes age over 60 years, elevated level of serum lactate dehydrogenase (LDH), Eastern Cooperative Oncology Group (ECOG) performance status, lymph nodes involvement, and extranodal sites of disease [13]. Our patient was in low risk category as per IPI with factor of elevated LDH.

Usually, the mainstay of therapy for large B cell non-Hodgkin lymphoma is the R-CHOP chemotherapy and role of radiation therapy (RT) as a consolidation therapy remains unclear. RT for SVCS is also not clearly defined but can be used for symptom relief after histologic diagnosis is made as it can obscure tissue biopsy results. In our case, the patient presented with SVCS as the mass was almost occupying the entire right lung. Since the patient was symptomatic RT was initiated for symptomatic relief after tissue biopsy, while waiting for immunohistochemistry report.

## 3. Conclusion

Although diffuse large B cell lymphoma subtype of primary pulmonary lymphoma is very rare, it should be included in the differential diagnosis of a lung mass with SVC syndrome. Because of its sporadic presentation and aggressive nature, the early recognition and prompt treatment are the key for outcome of the patient.
